# Invasive Functional Assessment of Coronary Artery Disease in Patients with Severe Aortic Stenosis in the TAVI Era

**DOI:** 10.3390/jcm12165414

**Published:** 2023-08-21

**Authors:** Maren Weferling, Won-Keun Kim

**Affiliations:** 1 Department of Cardiology, Kerckhoff Heart and Thorax Center, 61231 Bad Nauheim, Germany; w.kim@kerckhoff-klinik.de; 2 German Center for Cardiovascular Research (DZHK), Partnersite Rhein-Main, 10785 Berlin, Germany; 3 Department of Cardio-Thoracic Surgery, Kerckhoff Heart and Thorax Center, 61231 Bad Nauheim, Germany

**Keywords:** TAVI, coronary angiography, fractional flow reserve, coronary flow reserve, coronary artery disease

## Abstract

Coronary artery disease (CAD) is a common finding in patients suffering from aortic valve stenosis (AS), with a prevalence of over 50% in patients 70 years of age or older. Transcatheter aortic valve intervention (TAVI) is the standard treatment option for patients with severe AS and at least 75 years of age. Current guidelines recommend percutaneous coronary intervention (PCI) in patients planned for TAVI with stenoses of >70% in the proximal segments of non-left main coronary arteries and in >50% of left main stenoses. While the guidelines on myocardial revascularization clearly recommend functional assessment of coronary artery stenoses of less than 90% in the absence of non-invasive ischemia testing, a statement regarding invasive functional testing in AS patients with concomitant CAD is lacking in the recently published guideline on the management of valvular heart disease. This review aims to provide an overview of the hemodynamic background in AS patients, discusses and summarizes the current evidence of invasive functional testing in patients with severe AS, and gives a future perspective on the ongoing trials on that topic.

## 1. Introduction

Degenerative aortic valve stenosis is the most common valve disorder in Western countries, and its prevalence is rising with the increasing age of the population [1]. In patients over 70 years of age who suffer from severe aortic stenosis (AS), coronary artery disease (CAD) is prevalent in over 50% of cases, and both entities are associated with the presence of cardiovascular risk factors, such as smoking, hypertension, diabetes, and high low-density lipoprotein (LDL) levels, as potential etiological parameters [2,3,4]. Current European guidelines recommend coronary artery bypass grafting as an add-on procedure in cases with more than 70% stenosis in non-left main artery (and >50% left main stenosis) in AS patients planned for surgical aortic valve replacement (SAVR) with a class I recommendation and in cases with non-left main stenosis of 50–70% with a class IIa recommendation. When transcatheter aortic valve implantation (TAVI) is planned, percutaneous coronary intervention (PCI) is recommended in patients with more than 70% stenosis in the proximal segments of the coronary artery [5]. However, the evidence for these recommendations is poor and relies solely on angiographic assessment of epicardial stenoses. Furthermore, the optimal timing of PCI (either before, during, or after TAVI) remains to be elucidated. In the recently published European Society of Cardiology (ESC) consensus paper on CAD management in patients undergoing TAVI PCI *before* TAVI is recommended in stenoses >70% only in proximal segments in non-left main coronary arteries, particularly when angina pectoris or acute coronary syndrome or subtotal stenoses (i.e., >90%) are present [6]. Conversely, in the multicenter REVASC-TAVI registry comprising 1603 patients with stable CAD that assessed the outcome (all-cause mortality at two years) regarding PCI timing either before, concomitant with or after TAVI, two-year all-cause mortality was significantly lower when PCI was performed after TAVI compared with timing before or concomitant with TAVI (6.8% vs. 20.1% vs. 20.6%, respectively; *p* < 0.001) [7]. In general, the timing of PCI should be dependent on the clinical presentation, the anatomical characteristics, and the coronary lesion complexity of the patient [7].

While the current guideline for chronic coronary syndrome recommends invasive functional evaluation in all cases of coronary stenoses that do not exceed 90% in the absence of non-invasive ischemia testing [8], a statement regarding the role of functional evaluation of CAD in patients with aortic stenosis in the most recently published guideline on management of valve diseases [9] is lacking. One major reason for this might be a persisting uncertainty concerning the validity and reliability of intracoronary pressure assessment in this special patient subset due to the altered hemodynamic setting, which could potentially interfere with the functional testing. Currently, there are no data from randomized studies and only limited data from observational studies addressing this topic, and the consensus paper on CAD management in TAVI patients clearly states that further research in that field is needed [6]

The aim of this review is to provide an overview of the hemodynamic pathophysiology in AS patients, to summarize the available evidence on functional evaluation in coronary arteries in these patients, both with hyperemic and non-hyperemic indices, and to present a future perspective on ongoing trials dealing with this topic.

## 2. Functional Assessment of the Coronary Arteries—Basic Principles and Parameters

It is widely known that the mere angiographic assessment of coronary artery stenosis cannot predict its functional impact on coronary perfusion [10,11]. Therefore, over three decades ago, the calculation of fractional flow reserve (FFR) was introduced as an invasive method for the functional evaluation of coronary artery stenosis. The basic principle relies on the measurement of the coronary pressure distal (Pd) and proximal (Pa) to the stenosis, and a validated cut-off value of 0.8 was established for the ratio Pd/Pa. As myocardial perfusion is determined by coronary flow, and under normal circumstances, the latter is independent of coronary artery pressure due to autoregulatory mechanisms, it is necessary to perform the FFR measurement under conditions where coronary flow is directly dependent on coronary pressure. For this purpose, hyperemic substances, such as adenosine, are applied, that result in maximal hyperemia and subsequent abolishment of coronary autoregulation. Under such conditions, coronary flow is hypothetically linear and proportional to coronary pressure [12].

As adenosine has potential side effects, such as bradycardia, chest discomfort, and/or dyspnea, in more recent years, non-hyperemic pressure indices for functional assessment of coronary artery stenoses were introduced to overcome this issue. The instantaneous wave-free ratio (iFR) is a pressure-derived invasive index parameter registering the pressure in the so-called “wave-free” period during diastole. During this period, coronary microvascular resistance is lowest, and coronary flow and pressure are linearly related [13]. The optimal cut-off value of iFR that correlates best with a pathological FFR value <0.8 is 0.89, with an accuracy of 80% [14]. iFR has been proven to be non-inferior to FFR in terms of one-year major adverse cardiac events in two randomized-controlled trials [15,16]. Recently, further non-hyperemic pressure indices have been introduced, such as resting Pd/Pa, resting full-cycle ratio (RFR), and diastolic pressure ratio (dPR), that have been shown to correlate well with the gold-standard method FFR in observational trials and registries [17]. However, larger randomized controlled trials are still lacking.

Coronary flow reserve (CFR) is defined as the ratio of coronary flow under maximal vasodilation divided by coronary flow during resting conditions [18] and reflects the maximal capability of the coronary circulation to increase its blood supply according to elevated demand. It can be measured invasively by either the thermodilution-derived method with a dedicated pressure wire equipped with a temperature sensor at its tip or by the Doppler technique [19,20]. A CFR value of less than 2.0 is considered abnormal and can be due to either epicardial stenosis or microvascular disease or both [18]. In cases with a pathological CFR value in the absence of epicardial coronary artery disease, microvascular dysfunction can be presumed. Non-randomized studies have shown that the lower the CFR value is, the higher the adverse event rate [21,22].

The index of microvascular resistance (IMR) is derived from the pressure measured at the distal part of the coronary artery divided by flow. Based on studies measuring IMR in a healthy study population, the normal value is below 25 [23,24,25]. In contrast to CFR, IMR is not affected by epicardial stenoses and is solely a parameter of microvascular function.

### 2.1. Myocardial and Coronary Hemodynamics in Aortic Valve Stenosis—Pathophysiological Background

In patients with severe aortic stenosis, left ventricular (LV) afterload is typically increased due to the diminished aortic valve area, leading to elevated LV wall stress. As afterload is defined as the product of LV pressure and LV radius divided by LV wall thickness, concentric myocardial hypertrophy is the key mechanism to compensate for the pressure overload and hence increased afterload by normalizing LV wall stress and preserving cardiac output and LV ejection fraction [26,27,28]. However, LV hypertrophy leads to increased myocardial oxygen demand, causing ischemia and subsequent angina when it exceeds oxygen supply, even in the absence of obstructive coronary artery disease [26]. The onset of angina in AS patients is associated with worse outcomes [29,30]. In addition, increased LV end-diastolic filling pressure leads to compression of the microvasculature, particularly in the subendocardial area, thereby reducing coronary blood flow (CBF), most notably during exercise or tachycardia. At rest, CBF is typically sustained for a long course of time during disease progression due to the vasodilatory capacity of the intramyocardial vessels through autoregulatory mechanisms. The reduction of CBF during exertion is reflected by a decline in the CFR [31]. The impaired CFR is a consequence of elevated end-diastolic filling pressures, leading to the aforementioned compression of the microvasculature, arteriolar remodeling, perivascular fibrosis, and a relative reduction of the capillary density in the hypertrophied myocardium [32,33]. In addition, diastolic filling time and the severity of the aortic valve area have been positively correlated to reduced CFR [34]. Thus, even in the absence of epicardial CAD, ischemia can occur, further triggering fibrotic changes in the myocardium and inducing angina [35]. Due to these factors, the presence of angina has a low positive predictive value for CAD in patients with aortic stenosis [2]. The reduction of CFR in AS patients is associated with adverse long-term cardiovascular events [36,37].

### 2.2. Changes in Coronary Hemodynamics after TAVI

The immediate repair of AS by TAVI leads to an acute reduction of afterload and hence LV wall stress with subsequent decompression, especially of the subendocardial vasculature. Several studies investigated the acute changes in coronary hemodynamics in patients with AS immediately after TAVI, both in the presence and absence of CAD. However, these studies yielded conflicting results. Wiegernick et al. assessed intracoronary pressures and flow velocities using the Doppler-derived method in 27 AS patients before and directly after TAVI and compared them with 28 controls without AS [38]. The measurements were undertaken in non-obstructed left coronary arteries. They found an increase in hyperemic flow velocities and a decrease in hyperemic microvascular resistance and hence an increase in coronary flow velocity reserve post-TAVI by 10%, whereas the resting flow velocities and resistances remained unchanged [38]. Of note, the resting flow velocities were higher, and the resting resistances were lower in AS patients, leading to overall lower CFR values in AS patients compared with the controls. The observation of unchanged resting flow velocities in TAVI patients was attributed to the presence of LV hypertrophy with relative vessel rarefication and subsequent compensatory microvascular vasodilatation, a condition that is not resolved immediately after TAVI as regression of LV hypertrophy typically takes months or even years [38]. However, the comparison between the AS group and the control cohort must be interpreted with caution due to disparate baseline characteristics regarding age and sex distribution [38]. Nevertheless, the study findings underscore that TAVI has an immediate effect on coronary hemodynamics that is documented by a significant and prompt improvement in CFR, most likely due to immediate afterload reduction and subsequent decompression, particularly of the subendocardial myocardium. However, as the pathological resting flow velocities remain unaltered, TAVI does not directly lead to complete restoration of microvascular function, and, although not investigated in this study, it is most likely that this effect can be only expected after a certain amount of time has passed and LV hypertrophy has regressed.

Conversely, Camuglia et al. did not find an improvement in CFR assessed immediately after TAVI via a Doppler-tipped coronary wire in ten patients with high-grade AS with no relevant CAD on visual assessment (defined as the absence of epicardial coronary stenoses >30%): The mean change in CFR immediately post-TAVI vs. pre-TAVI was 0.045 (95% CI: 0.4 to 0.49; *p* = 0.41), although, at the 12-month follow-up, a significant increase in CFR was detected (mean absolute increase: 0.65 [95%CI 0.36–0.9]; *p* < 0.01) [39]. Stoller et al. assessed CFR, FFR, and coronary collateral flow index (CFI) in 40 patients with severe AS pre-TAVI and immediately post-TAVI [40]. Twenty-six patients had CAD (defined as >50% lumen reduction), and the rest had no CAD. The authors did not find a significant change in CFR post-procedurally in either of the groups [40]. In contrast to Wiegernick et al., the authors used the thermodilution method to assess CFR instead of a Doppler-based method. Hence, the authors assume that the reason for these conflicting results is, firstly, that the Doppler-based method might be more “robust” in assessing CFR than the thermodilution method and, secondly, that the presence of CAD in their cohort might have influenced the CFR values [40].

In a recently published study, Wada et al. performed serial Doppler-derived measurements (via echocardiography) of CFR before TAVI, one day after TAVI, and at the 12-month follow-up in 59 patients with severe AS [41]. While CFR was impaired in 59% of patients at baseline (median CFR 1.75 [IQR 1.5–2.1]), a reduced CFR was still present in 39% of patients one day after TAVI and in only 4% after 12 months (*p* < 0.001) [41]. The median CFR value increased from 1.75 pre-TAVI to over 2.0 one day post-TAVI. One year after TAVI, it was 2.6. Patients with a greater increase in CFR (defined as a median value of 0.9 or more) had a greater aortic valve area (AVA) and lower mean aortic transvalvular pressures post-TAVI and a lower pre-TAVI LV ejection fraction with greater improvement at the one-year follow-up. The improvement in CFR was driven by an increase in hyperemic flow, whereas the resting flow velocities remained unchanged at each time point [41]. These results are in line with the findings by Wiegernick et al., who also found a direct increase in CFR immediately after TAVI that was due to an increase in hyperemic flow and unaltered resting flow velocities [38].

Vendrik et al. investigated resting and hyperemic coronary flow velocities via an invasive Doppler-based approach in 13 AS patients pre-TAVI, immediately after TAVI, and at a six-month follow-up [42]. Like Wada et al., they detected an immediate increase in CFR directly after TAVI that further improved after six months (1.28 [1.10–1.85] pre-TAVI, 1.65 [1.47–1.85] post-TAVI, 1.94 [1.69–2.25] after six months). Consistent with the aforementioned studies, the CFR improvement was a result of an increased hyperemic flow, whereas resting flow velocities were unchanged [42]. Conversely, Sabbah et al., who investigated CFR (via the thermodilution method) and FFR values in 34 patients with AS undergoing transcatheter (17 patients) and surgical aortic valve replacement (17 patients), found an increase in CFR after six months that was not related to a rise in hyperemic flow but to a decrease in resting flow [43]. The authors concluded that the reduced resting flow was due to a diminished myocardial oxygen demand as a consequence of the reduced stroke work. Hyperemic flow measurements were not altered [43].

In summary, the majority of studies investigating the effect of TAVI on coronary hemodynamics found an immediate improvement in CFR due to increased hyperemic flow, while the resting flow remained unchanged. This finding was even more pronounced after a certain amount of time had passed, most likely due to regression of LV hypertrophy and the accompanying morphological changes, such as extravascular decompression of the subendocardial microvasculature and remodeling processes. As a limitation, all studies have in common that the sample sizes were small and that a comparison with a matched control cohort was seldom performed. Thus, the results must be interpreted with caution.

### 2.3. Functional Assessment of Epicardial Coronary Stenosis Using FFR

The current European guidelines recommend invasive functional assessment of coronary artery stenoses below 90% with a class IA grading in the absence of non-invasive testing for ischemia [5]. This recommendation is based on the evidence of large clinical trials, such as the FAME studies that compared angiographic versus FFR guidance for treatment decision-making in intermediate coronary artery stenoses [44] and deferral (with optimal medical therapy) versus PCI of coronary stenoses with pathological FFR values [45]. The results showed a clear benefit of FFR in terms of the composite primary outcome measure death, myocardial infarction, and urgent revascularization. However, all of these trials included primarily patients with stable CAD or myocardial infarction (with functional assessment of non-culprit lesions) but not patients with severe AS. In addition, patients with significant valve disease were excluded from the initial validation of FFR [11,46]. Hence, it is not clear whether the recommendations and cut-off values from the invasive functional assessment can be applied to patients with severe AS. From a pathophysiological perspective, because LV hypertrophy leads to external compression of the microvasculature and relative capillary rarefication, the resting vasodilatory capacity to maintain stable coronary flow is already exhausted in AS patients [38,47]. Thus, the vasodilatory response to adenosine is attenuated during FFR measurement, which may lead to false negative (too high) FFR values in intermediate epicardial stenoses and subsequent underestimation of stenosis severity [35]. Structural and functional alterations of the microvasculature further contribute to this finding, including perivascular fibrosis, arteriolar remodeling, endothelial dysfunction, and a higher sympathetic tone, all of which lead to a higher hyperemic microvascular resistance with subsequent attenuation of the vasodilatory effect of adenosine [34,38,47,48].

With this pathophysiology as a background, the study by Vendrik et al. mentioned above assessed FFR and iFR in the cohort of 13 patients with intermediate- to high-grade coronary stenoses [42]. FFR values decreased directly post-TAVI compared with baseline and decreased further at six months follow-up: 0.85 (0.76–0.88) pre-TAVI to 0.79 (0.74–0.83) post-TAVI and then to 0.71 (0.65–0.77) after six months (*p* < 0.001 for all comparisons). This decline was primarily due to increased hyperemic coronary flow velocities and underscores that the vasodilatory capacity of the microvasculature improves in the short term, most probably due to the immediate reduction of afterload right after TAVI, and in the long term, most likely as a result of LV hypertrophy regression and favorable LV remodeling, which is usually a process lasting weeks to months after aortic valve replacement [42]. The limitations of this study are the small sample size (n = 13) and the fact that adenosine was administered as an intracoronary bolus, as the use of intravenous adenosine is much more common and might yield different results. However, other studies support the findings by Vendrik et al., at least considering the short-term effect of TAVI on hemodynamics. Ahmad et al. assessed FFR values and flow parameters (among other functional indices) in 28 patients with severe AS and concomitant moderate or severe CAD [49]. They also found a decline in FFR values immediately after TAVI: FFR 0.87 +/−0.08 pre-TAVI vs. 0.85 +/− 0.09 post-TAVI (*p* = 0.0008). This effect was due to the fact that hyperemic systolic flow increased significantly post-TAVI [49]. Pesarini et al. measured FFR values before and immediately after TAVI in 54 patients with concomitant intermediate CAD [50]. Overall, the FFR values only changed mildly, however, considering the lesions with positive FFR values (<0.80; 16% of all patients), a significant further reduction in FFR was detected right after TAVI, whereas lesions with negative FFR values tended to improve post-TAVI. The treatment decision based on the FFR cut-off of 0.80 changed after TAVI in only 6% of all lesions [50]. Similarly, Stundl et al. performed FFR measurements in 31 patients with coronary stenosis diameter of 50% or greater before TAVI. In the case of a pathological FFR value, the lesion was not treated before TAVI [51]. In 12 of these patients, the FFR measurement was repeated six to eight weeks after TAVI. A pairwise analysis of the pre- and post-TAVI FFR values revealed no relevant differences [51]. In a recently published retrospective analysis, FFR-guided PCI was found to be associated with a better outcome (major adverse cardiovascular and cerebrovascular event (MACCE)-free survival at 24 months) compared with angiography-guided PCI in 216 AS patients undergoing TAVI [52]. Limitations, however, must be considered in that it was an observational retrospective analysis and thus was potentially susceptible to possible confounders and that in both groups, PCI was either performed pre-TAVI, peri-interventionally during TAVI, or post-TAVI. Thus, it is not clear whether pre-TAVI PCI is disadvantageous compared with PCI after valve implantation. Table 1 gives a summary of studies investigating the benefits of FFR pre- and post-TAVI.

In summary, the majority of studies investigating FFR changes in AS patients after TAVI show a decline in FFR values immediately after TAVI that is further aggravated months after TAVI. From a clinical standpoint, these findings indicate that FFR measurements of intermediate coronary artery stenoses should be performed preferentially after TAVI, ideally after a certain period of time when regression of LV hypertrophy has occurred, especially in lesions with borderline significance.

### 2.4. Functional Assessment of Epicardial Coronary Stenosis Using Non-Hyperemic Indices

In several studies, invasive functional assessment of moderate or severe epicardial stenoses was performed, and the changes pre- vs. post-TAVI in non-hyperemic indices were compared with the gold-standard FFR [42,49,53]. In the cohort of Ahmad et al. of 28 AS patients with moderate or severe CAD mentioned above, the authors found that iFR values did not change after TAVI, whereas FFR values decreased significantly. The latter result was driven by an increased hyperemic flow, especially during systole, whereas resting flow measurements did not change. The authors concluded that non-hyperemic indices such as iFR should be preferred for functional evaluation of CAD in AS patients since these parameters are not affected by AS and thus are less vulnerable to changes during TAVI [49]. Conversely, in 66 patients with severe AS, Scarsini et al. found that although the mean iFR values were not different before and after TAVI (0.89 ± 0.12 vs. 0.89 ± 0.12, *p* = 0.66), there were wide individual variations in the values that led to a crossing of the threshold of 0.89 in 15% of lesions and hence a change in the treatment decision, especially in patients with more severe aortic valve gradients and higher transaortic pressure drops after TAVI [53]. These authors found a high negative predictive value for the iFR cut-off of 0.89 to exclude functionally significant coronary lesions but a low positive predictive value for detection of functionally relevant lesions in both pre- and post-TAVI settings when correlated with the gold-standard cut-off FFR of 0.80 [53]. Hence, the authors conclude that the iFR cut-off of 0.89 might not be appropriate in AS patients, and a threshold lower than 0.89 might be more appropriate [53]. However, given the hypothesis derived from the aforementioned studies that FFR might underestimate the functional relevance of coronary stenoses due to attenuations in hyperemic coronary flow, which would not hold true for iFR measurements, it may be more reasonable to adhere to the conventional iFR cut-off of 0.89 and apply a cut-off higher than 0.80 for FFR in this setting. A study by Yamanaka et al., on the other hand, questioned the validity of the conventional iFR cut-off in AS patients by finding that a shift of the iFR cut-off value from 0.89 to 0.82 correlated better with proven myocardial ischemia in myocardial perfusion scintigraphy tested in 95 AS patients with concomitant intermediate coronary stenoses [54]. Similarly, Scarsini et al. correlated FFR and iFR measurements with the results of non-invasive stress single-photon computed tomography (SPECT) in 28 patients with severe AS and borderline CAD [55]. While FFR showed a very good agreement with ischemia detected by SPECT, this was not the case for iFR: The conventional cut-off value of 0.89 (iFR) led to a significantly higher false-positive rate (i.e., iFR < 0.89, negative SPECT) compared with the FFR cut-off of 0.80 (39% vs. 12%, *p* = 0.011) [55]. In a study by Kleczynski et al. with a larger sample size (416 lesions in 221 patients with severe AS and concomitant CAD), the overall diagnostic accuracy of FFR in detecting lesions with an iFR ≤ 0.89 was 0.997 (95%CI 0.986 to 1.000; *p* < 0.001), and, conversely, for iFR to detect lesions with an FFR ≤ 0.80 this was 0.995 (95%CI 0.983 to 0.999; *p* < 0.001) [56]. However, they found that the optimal cut-off value for FFR to detect an iFR value of 0.89 was 0.82 (sensitivity, specificity, and accuracy of 97.1%, 98.9%, and 97.7%, respectively), and for iFR to detect an FFR value of 0.80, this cut-off was 0.88 (sensitivity, specificity, and accuracy of 99.1%, 95.8%, and 97.4%, respectively) [56].

In summary, studies indicate that iFR values are less affected by TAVI than FFR measurements due to the mostly unaltered flow during the diastolic wave-free period before and after TAVI. The validity of the iFR cut-off of 0.89, however, remains controversial, as compared with both the gold-standard FFR and non-invasive ischemia testing, more false-positive lesions were detected that could potentially result in unnecessary coronary interventions. Even the usefulness of the standard cut-off value of FFR in the setting of AS has to be discussed, as several studies indicate that hyperemic flow is attenuated, leading to higher and, thus, potentially false-negative FFR values. Of note, all studies have in common that the sample sizes were small, and the extent of concomitant CAD differed across the studies.

## 3. Conclusions and Future Perspectives

Our current knowledge of the role of invasive functional testing with either hyperemic or non-hyperemic pressure indices in AS patients with concomitant CAD before and after TAVI is based on retrospective, observational analyses of small numbers of patients. Therefore, the conclusions drawn from these studies are mainly hypothesis-generating. In order to adequately address issues such as the validity of the conventional cut-off values of FFR and iFR in AS patients, the effect of these cut-off values on outcome in AS patients undergoing TAVI, and the best timepoint for invasive functional evaluation of CAD in AS patients, larger-scaled studies carried out in a prospective, randomized fashion are urgently needed.

Currently, there are several ongoing trials addressing the aforementioned topics that will hopefully provide answers to these unresolved questions in the future. One such trial is the FAITAVI study (NCT03360591), a prospective, randomized, open-label, multicenter trial with planned enrollment of 320 AS patients undergoing TAVI that will compare an angiography-guided vs. a physiology-guided PCI strategy (both FFR and iFR) with regard to 12-month primary outcome (composite of all-cause death, myocardial infarction, stroke, major bleeding, and need for target vessel revascularization). Additionally, in a post-hoc analysis, different iFR cut-off values are planned to be compared with the standard cut-off of 0.80 in FFR measurements in terms of the same endpoints. The NOTION-3 (NCT0305862) trial is designed to compare TAVI only vs. TAVI + FFR-guided PCI with regard to the primary endpoints all-cause mortality, myocardial infarction, or urgent revascularization within a 12-month follow-up with a planned recruitment of 454 participants. The TAVI-PCI trial (NCT04310046) will investigate differences between a complete revascularization strategy via PCI before and after TAVI in stenoses >90% or with a pathological iFR measurement in terms of the primary outcome measures all-cause death, non-fatal myocardial infarction, ischemia-driven revascularization, rehospitalization (valve- or procedure-related including heart failure), and life-threatening/disabling or major bleeding (according to VARC-2). Of note, in the “PCI after TAVI” arm, iFR measurements are made both before and after TAVI and before PCI.

## Figures and Tables

**Table 1:** Studies investigating hyperemic indices in AS patients with concomitant CAD undergoing TAVI.

References	Year	Study Design	n	Results	*p*-Values
Vendrik et al. [42]	2020	Prospective, observational; FFR and iFR measurements before TAVI, immediately after TAVI and at six-month FU	13	FFR values decreased from 0.85 (0.76–0.88) pre-TAVI to 0.79 (0.74–0.83) post-TAVI and 0.71 (0.65–0.77) at six-month FU; iFR values did not change: 0.82 (0.80–0.90) pre-TAVI, 0.83 (0.77–0.88) post-TAVI, and 0.83 (0.73–0.89) at six months	FFR before vs. after TAVI: *p* < 0.001; iFR: *p* = 0.735
Ahmad al. [47]	2019	Prospective, observational; assessment of microvascular function, FFR and iFR in both AS and no AS pts with concomitant CAD before and immediately after TAVI; in no AS pts before and after PCI	55 (AS pts)	FFR values decreased from 0.86 (±0.08) pre-TAVI to 0.83 (±0.09) post-TAVI; iFR values did not change: 0.87 (±0.10) pre-TAVI and 0.87 (±0.09) post-TAVI	FFR before vs. after TAVI: *p* < 0.001 iFR: *p* = 0.80
Ahmad et al. [49]	2018	Prospective, observational; intracoronary flow-, FFR and iFR measurements immediately before and after TAVI	28	FFR values changed from 0.87 ± 0.08 pre-TAVI to 0.85 ± 0.09 post-TAVI; iFR values did not change: 0.88 ± 0.09 pre-TAVR vs. 0.88 ± 0.09 post-TAVR	FFR before vs. after TAVI: *p* < 0.001; iFR: *p* = 0.73
Pesarini et al. [50]	2016	Prospective, observational; FFR measurements before and after TAVI during the same procedure	54	No relevant FFR change before and after TAVI: 0.89 ± 0.10 vs. 0.89 ± 0.13; however, FFR values in >50% stenosis worsened after TAVI 0.84 ± 0.12 vs. 0.82 ± 0.16, whereas FFR in mild stenosis (<50%) improved slightly: 0.90 ± 0.07 vs. 0.91 ± 0.09	Overall FFR change before and after TAVI: *p* = 0.73; FFR values in >50% stenosis before vs. after TAVI: *p* = 0.02; FFR values in <50% stenosis: *p* = 0.69
Stoller et al. [40]	2018	Prospective, observational; FFR and CFR measurements before and after TAVI	40	Improvement of FFR values after TAVI compared with FFR before TAVI: 0.90 ± 0.08 vs. 0.93 ± 0.08; CFR values did not change significantly: 1.9 ± 0.09 vs. 2.0 ± 1.0	FFR before vs. after TAVI: *p* = 0.0021; CFR before vs. after TAVI: *p* = 0.72
Lunardi et al. [52]	2019	Retrospective; comparison of FFR- vs. angiography guidance in patients with AS and concomitant CAD planned for TAVI in terms of MACCE at two-year FU	216	MACCE-free survival was better in the FFR-guided group versus angio-guided group: 92.6% vs. 82.0%; hazard ratio 0.4; 95% CI 0.2–1.0	*p* = 0.035
Stundl et al. [51]	2020	Prospective observational; FFR measurements in coronary artery stenosis >50% in AS pts before and six to eight weeks after TAVI	13	No relevant difference in FFR values before and after TAVI: 0.77 ± 0.04 vs. 0.76 ± 0.08	*p* = 0.11

Abbreviations: FFR = fractional flow reserve; iFR = instantaneous wave-free ratio; FU = follow-up; TAVI = transcatheter aortic valve intervention; MACCE = major adverse cardiac and cerebrovascular events; pts = patients.

## Data Availability

Not applicable.
